# Synthesis, crystal structure and Hirshfeld and thermal analysis of bis[benzyl 2-(heptan-4-yl­idene)hydrazine-1-carboxyl­ate-κ^2^ *N* ^2^,*O*]bis(thio­cyanato)­nickel(II)

**DOI:** 10.1107/S2056989020004260

**Published:** 2020-04-07

**Authors:** Palanivelu Nithya, Subbiah Govindarajan, Jim Simpson

**Affiliations:** a Department of Chemistry, Bharathiar University, Coimbatore - 641 046, Tamil Nadu, India; b Department of Chemistry, J. J. College of Arts and Science, Pudukkottai, - 622 422, Tamil Nada, India; c Department of Chemistry, University of Otago, PO Box 56, Dunedin 9054, New Zealand

**Keywords:** crystal structure, Ni^II^ complex, benzyl-2-(heptan-4-yl­idene)hydrazine-1-carboxyl­ate ligand, thio­cyanato ligands, Hirshfeld surface analysis, simultaneous TGA–DTA analyses

## Abstract

The title centrosymmetric Ni^II^ complex, [Ni(NCS)_2_(C_15_H_22_N_2_O_2_)_2_], crystallizes with one half mol­ecule in the asymmetric unit of the monoclinic unit cell. The complex adopts an octa­hedral coordination geometry with two mutually *trans* benzyl-2-(heptan-4-yl­idene)hydrazine-1-carboxyl­ate ligands in the equatorial plane with the axial positions occupied by N-bound thio­cyanato ligands. The overall conformation of the mol­ecule is also affected by two, inversion-related, intra­molecular C—H⋯O hydrogen bonds. The crystal structure features N—H⋯S, C—H⋯S and C—H⋯N hydrogen bonds together with C—H⋯π contacts that stack the complexes along the *b-*axis direction. The packing was further explored by Hirshfeld surface analysis. The thermal properties of the complex were also investigated by simultaneous TGA–DTA analyses.

## Abstract 2

The mol­ecular and crystal structure of bis­[benzyl 2-(heptan-4-yl­idene)hydrazine-1-carboxyl­ate-κ^2^ *N* ^2^,*O*]bis­(thio­cyanato)­nickel(II) is reported. Hirshfeld surface and simultaneous TGA–DTA analyses are also described

## Chemical context   

Investigations of the Schiff base complexes of benzyl carbazate are scarce except for our own reports (Nithya *et al.*, 2016 ▸, 2017*a* ▸,*b* ▸, 2018*a* ▸,*b* ▸). These complexes are formed by Schiff base carbazate ligands in their keto form with *N*,*O* chelation to give complexes with octa­hedral geometry. The coordination chemistry of benzyl carbazate Schiff base complexes has gained importance not only from the inorganic point of view, but also because of their biological and thermal properties. In the course of our recent studies on such complexes, we reported the cobalt(II) complex of a Schiff base derived from benzyl carbazate and heptan-4-one with thio­cyanates as the charge-compensating ligands (Nithya *et al.*, 2019 ▸). In this work, we report the synthesis, mol­ecular and crystal structures, Hirshfeld surface analysis and thermal properties of the corresponding nickel complex, bis­[benzyl-2-(heptan-4-yl­idene)hydrazine-1-carboxyl­ate]bis­(thio­cyanato)­nickel(II), **1**.
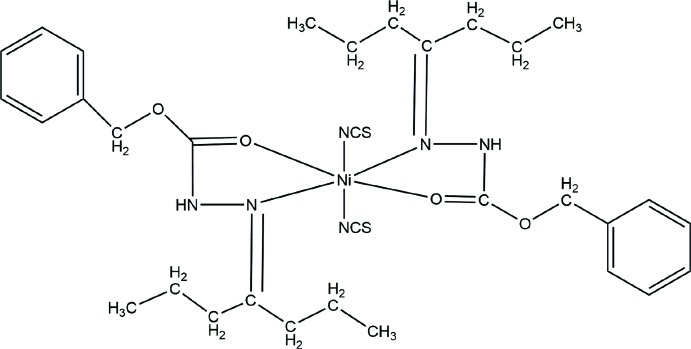


## Structural commentary   

**Figure 1:**
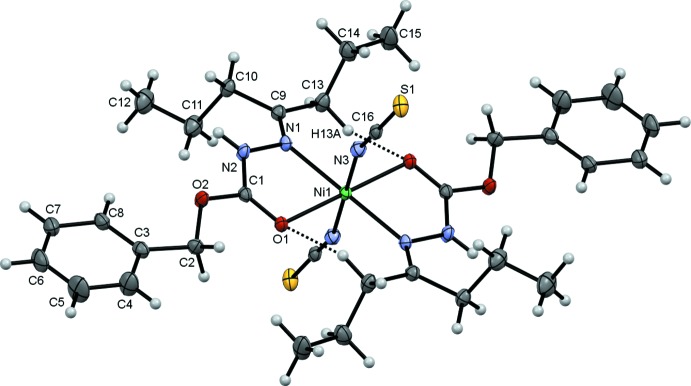
The mol­ecular structure of **1** showing the atom numbering with ellipsoids drawn at the 50% probability level. Labelled atoms are related to unlabelled atoms by the symmetry operation −*x* + 1, −*y*, −*z* + 2. Intra­molecular hydrogen bonds are shown as dashed black lines.

The title compound, **1**, crystallizes in the space group *P*2_1_/*c* with one half of the complex in the asymmetric unit as the Ni^II^ cation lies on an inversion centre, Fig. 1 ▸. This contrasts with the previously determined Co^II^ analogue (Nithya *et al.*, 2019 ▸) that crystallizes with two unique, centrosymmetric complex mol­ecules in the asymmetric unit. Two inversion-related intra­molecular C13—H13*A*⋯O1 hydrogen bonds, Table 1 ▸, influence the conformation of the benzyl-2-(heptan-4-yl­idene)hydrazine-1-carboxyl­ate ligands and enclose 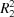
(14) ring motifs. Two hydrazine-carboxyl­ate ligands chelate the Ni atom with N1 and O1 donor atoms; these chelating ligands lie *trans* to one another in the equatorial plane of the slightly distorted octa­hedral complex. The axial positions are occupied by two thio­cyanato ligands bound to the metal through their N3 atoms. The NCS ligands are kinked away from the alkane chains of the other ligands with C16—N3—Ni1 angles of 163.23 (11)°. Bond lengths and angles in the closely related Ni and Co complexes are generally similar, although the Ni1—N1 bond [2.1332 (12) Å] is significantly shorter here than the corresponding Co1—N11 and Co2—N21 vectors [2.206 (5) and 2.248 (6) Å respectively].

## Supra­molecular features   

**Figure 2:**
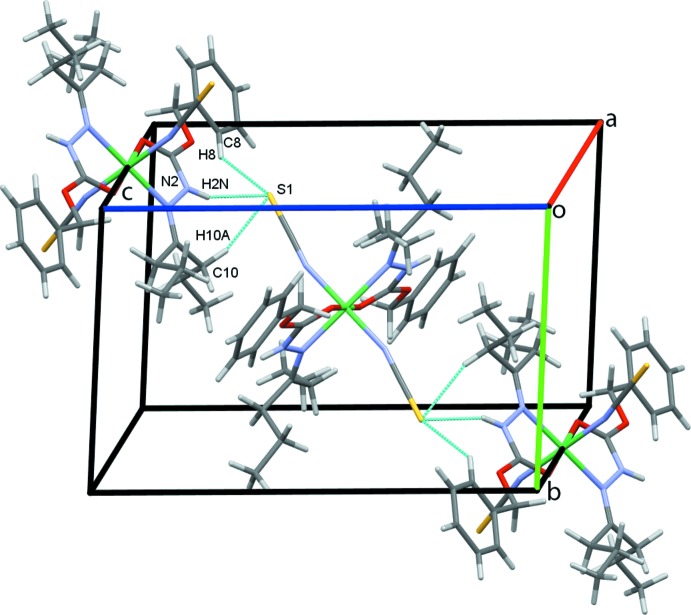
Chains of mol­ecules of **1** along the *bc* diagonal. Hydrogen bonds are drawn as dashed cyan lines.

**Figure 3:**
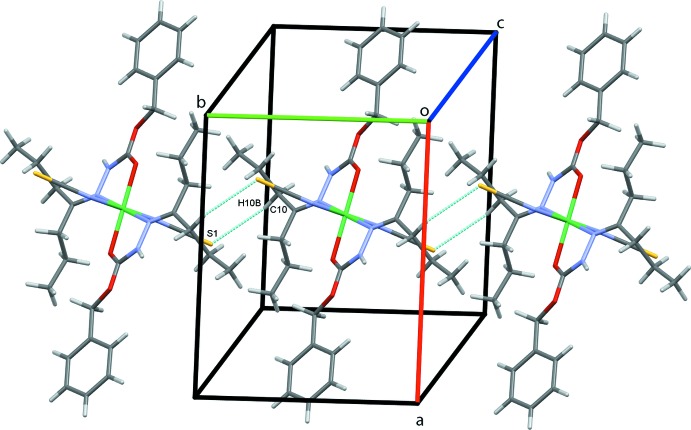
Chains of inversion dimers of **1** along *b*.

**Figure 4:**
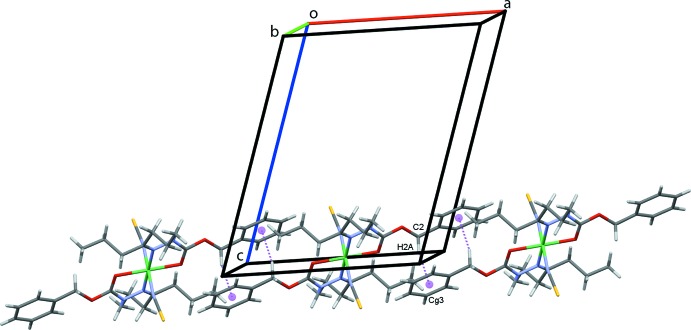
Chains of mol­ecules of **1** along *a*. C—H⋯π contacts are drawn as dashed magenta lines with the centroids (*Cg*) of the C3–C8 rings shown as magenta spheres.

**Figure 5:**
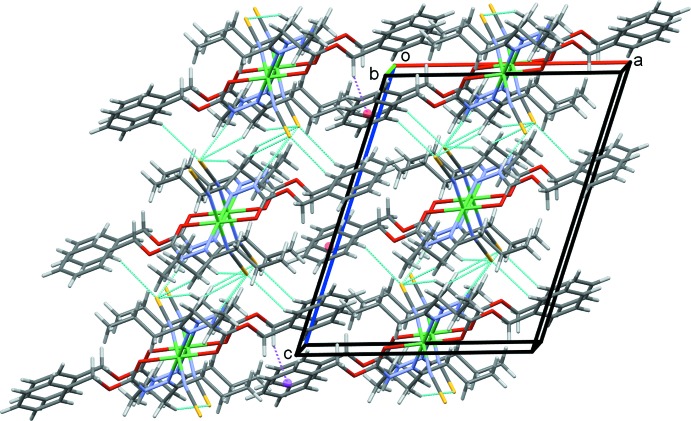
Overall packing of **1** viewed along the *b*-axis direction.

In the crystal structure, atom S1 acts as a trifurcated acceptor forming N2—H2*N*⋯S1 and weaker C8—H8⋯S1 and C10—H10*A*⋯S1 hydrogen bonds, Table 1 ▸, that form chains of complex mol­ecules along the *bc* diagonal, Fig. 2 ▸. Inversion-related pairs of C10—H10*B*⋯S1 hydrogen bonds link adjacent mol­ecules into rows along the *b-*axis direction, Fig. 3 ▸, while rows also form along *a*, through C2—H2*A*⋯*Cg*3, C—H⋯π contacts, Fig. 4 ▸; *Cg*3 is the centroid of the C3–C8 phenyl ring. These contacts combine to stack mol­ecules of the complex in a regular fashion along the *b*-axis direction, Fig. 5 ▸.

## Hirshfeld surface analysis   

**Figure 6:**
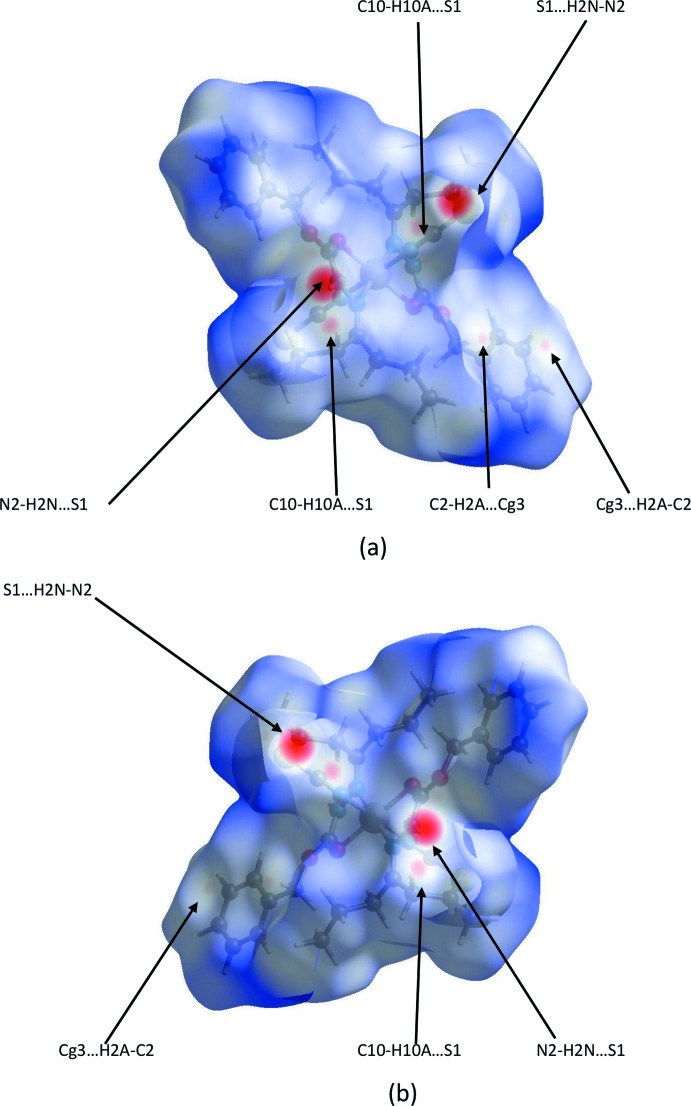
Hirshfeld surfaces for opposite faces (*a*) and (*b*) of **1** mapped over *d*_norm_ in the range −0.3928 to 2.1718 a.u. *Cg*3 is the centroid of the C3–C8 phenyl ring.

**Figure 7:**
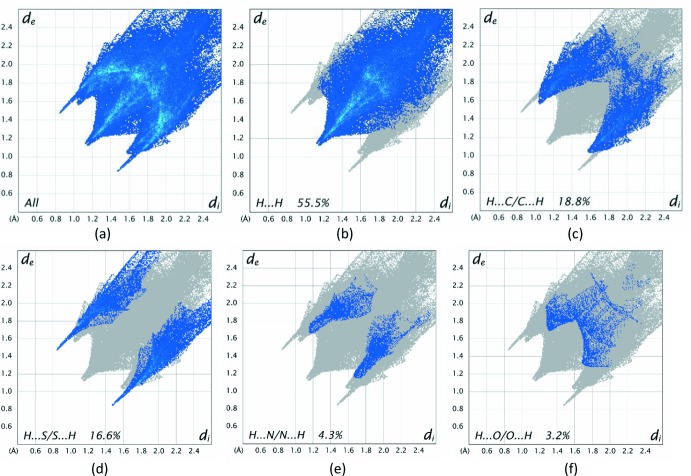
A full two-dimensional fingerprint plot for **1**, (*a*), together with separate principal contact types for the mol­ecule (*b*)–(*f*). These were found to be H⋯H, H⋯C/C⋯H, H⋯S/S⋯H, H⋯N/N⋯H and H⋯O/O⋯H contacts.

Further details of the inter­molecular inter­actions in **1** were obtained using Hirshfeld surface analysis (Spackman & Jayatilaka, 2009 ▸) with Hirshfeld surfaces and two-dimensional fingerprint plots generated with *CrystalExplorer17* (Turner *et al.*, 2017 ▸). Hirshfeld surfaces for opposite faces of **1** are shown in Fig. 6 ▸(*a*) and (*b*). Bold red circles on the Hirshfeld surfaces correspond to the N—H⋯S hydrogen bonds while the weaker C—H⋯S and C—H⋯π contacts appear as faint red circles. Fingerprint plots, Fig. 7 ▸, reveal that while H⋯H inter­actions make the greatest contributions to the surface contacts, as would be expected for a mol­ecule with such a predominance of H atoms, H⋯C/C⋯H and H⋯S/S⋯H contacts are also substantial, Table 2 ▸. H⋯N/N⋯H and H⋯O/O⋯H contacts are less significant, with the O⋯C/C⋯O and O⋯S/S⋯O contacts being essentially trivial with contributions of 0.7% and 0.6%, respectively. These are not shown in Fig. 7 ▸ but are included in Table 3 ▸ for completeness.

## Thermal properties   

**Figure 8:**
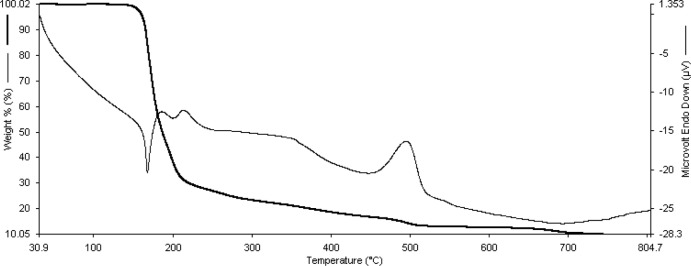
Simultaneous TGA–DTA analyses for **1**. The heavy (darker) lines show the TGA plot with the DTA behaviour shown by the lighter curve.

Fig. 8 ▸ shows the thermal decomposition behaviour of **1**. Simultaneous TGA–DTA analyses were recorded in air on a Perkin–Elmer SII Thermal Analyser over the temperature range 50–800°C. With the equipment used here, the TGA curve shows the temperature range but not the individual peak temperatures. However, peak temperatures can be seen in the DTA curve. In the first step of decomposition, the weight loss of 74% occurs over the temperature range 115–260°C (TGA). This corresponds to the loss of the Schiff base ligands to form Ni^II^ thio­cyanate as an inter­mediate. This was marked by both endothermic (170°C) and exothermic peaks (190 and 210°C) in the DTA curve. As the thermal analysis was carried out under a dynamic flowing air atmosphere, the S and N atoms are oxidized to SO_2_ and NO_2_, while nickel ultimately forms nickel oxide. Similar decomposition processes have been observed in our recent wok on numerous similar complexes, see for example (Nithya *et al.*, 2017*a* ▸,*b* ▸, 2018*a* ▸,*b* ▸, 2019*a* ▸,*b* ▸).

## Database survey   

As mentioned previously, the most closely related structure to the one reported here is that of the Co^II^ analogue (Nithya *et al.* 2019 ▸) while we have also reported the structures of 18 other Schiff base complexes of various transition metals with ligands based on benzyl carbazate (Nithya *et al.* 2016 ▸, 2017*a* ▸,*b* ▸, 2018*a* ▸,*b* ▸). A search in the Cambridge Structural Database (version 5.41, November 2019; Groom *et al.*, 2016 ▸) for other related transition-metal complexes produced no additional hits. The novelty of the ligands found in these complexes is reinforced by the fact that a search for organic compounds incorporating the PhCH_2_OC(O)NHN=C(CH_2_)_2_ unit produced only two hits. One was our own report of the ligand benzyl 2-cyclo­pentyl­idenehydrazine­carboxyl­ate (JENFAM; Nithya *et al.*, 2017*a* ▸). The other was (2*E*)-1-ethyl 8-methyl 7-(2-(benzyl­oxycarbon­yl)hydrazono)oct-2-enedioate, (VEWMOA; Gergely *et al.*, 2006 ▸). In both cases, the bond distances and angles in the structures compare very favourably with those reported here.

## Synthesis and crystallization   

Equimolar amounts of ammonium thio­cyanate (0.076 g, 1 mmol) and benzyl carbazate (0.166 g, 1 mmol) were dissolved in methanol (10 mL). Nickel nitrate, Ni(NO_3_)_2_·6H_2_O, (0.146 g, 0.5 mmol) dissolved in 10 mL of doubly distilled water was added to this solution. The resulting blue solution was layered with heptan-4-one (dipropyl ketone) and the solution changed to a green colour. The final solution was left to evaporate at room temperature. After slow evaporation, bluish–green rhombus-shaped crystals suitable for X-ray diffraction analysis were collected, washed with doubly distilled water and air-dried.

Analysis calculated for NiC_32_H_44_N_6_O_4_S_2_: Ni, 8.40; C, 54.96; H, 6.30; N, 12.02; S, 9.16%. Found: Ni, 8.25; C, 54.76; H, 6.13; N, 11.80; S, 9.08%; conductance = 14 *S* cm^2^ mol^−1^. Yield based on the metal: 80%.

The FT–IR spectrum was recorded on a JASCO-4100 FT–IR spectrophotometer from 4000 to 400 cm^−1^ using KBr pellets: N—H stretch 3152 cm^−1^ C=O stretch 1675 cm^−1^ C=N stretch 1524 cm^−1^, N—N stretch 1058 cm^−1^. 2108 cm^−1^ C≡N stretch of the N-bound thio­cyanate ligands.

The electronic absorption spectrum was measured on a JASCO V-630 UV–vis spectrophotometer and recorded in methanol at room temperature: intense bands at 392, 678 and 732 nm were assigned to the ^3^ *A*_2*g*_ → ^3^ *T*_2*g*_, 3*A*_2*g*_ → ^3^ *T*_1g_(*F*) and ^3^ *A*_2*g*_(*F*) →^3^ *T*_1*g*_(*P*) transitions, respectively, supporting the six-coordinate octa­hedral geometry around the Ni^II^ cation (Lever, 1984 ▸).

The ^1^H NMR spectrum was recorded on a Bruker AV 400 (400 MHz) spectrometer using tetra­methyl­silane as an inter­nal reference. Chemical shifts are expressed in parts per million (ppm): 0.84–0.88 and 1.33–2.20 ppm: CH_3_ and CH_2_ groups, respectively; –OCH_2_ proton: 5.08 ppm; aromatic protons multiplets 7.29–7.34 ppm; NH: 9.882 ppm.

Simultaneous TGA–DTA analyses were recorded in air on a PerkinElmer SII Thermal Analyser over the temperature range 50-800°C.

## Refinement   

Crystal data, data collection and structure refinement details are summarized in Table 3 ▸. The N—H hydrogen atom was located in a difference-Fourier map and its coordinates refined with *U*_iso_(H) = 1.2*U*_eq_(N). All C-bound H atoms were refined using a riding model with *d*(C—H) = 0.95 Å, *U*_iso_ = 1.2*U*_eq_(C) for aromatic 0.99 Å, *U*_iso_ = 1.2*U*_eq_(C) for CH_2_ and 0.98 Å, *U*_iso_ = 1.5*U*_eq_(C) for CH_3_ H atoms.

## Supplementary Material

Crystal structure: contains datablock(s) I, global. DOI: 10.1107/S2056989020004260/vm2230sup1.cif

Structure factors: contains datablock(s) I. DOI: 10.1107/S2056989020004260/vm2230Isup2.hkl

CCDC reference: 1993291

Additional supporting information:  crystallographic information; 3D view; checkCIF report

## Figures and Tables

**Table 1:** Hydrogen-bond geometry (Å, °) *Cg* is the centroid of the C3–C8 phenyl ring.

*D*—H⋯*A*	*D*—H	H⋯*A*	*D*⋯*A*	*D*—H⋯*A*
N2—H2*N*⋯S1^i^	0.824 (17)	2.507 (17)	3.2830 (12)	157.3 (16)
C8—H8⋯S1^i^	0.95	2.94	3.7080 (16)	139
C10—H10*A*⋯S1^i^	0.99	3.00	3.9059 (14)	154
C10—H10*B*⋯S1^ii^	0.99	2.94	3.8464 (15)	153
C13—H13*A*⋯O1^iii^	0.99	2.35	3.1783 (18)	141
C2—H2*A*⋯*Cg*3^iv^	0.99	2.72	3.6041 (17)	149

Symmetry codes: (i) 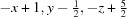; (ii) 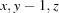; (iii) 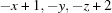; (iv) 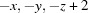.

**Table 2:** Percentage contributions to the Hirshfeld surface for **1**

Contacts	Included surface area %
H⋯H	55.5
H⋯C/C⋯H	18.8
H⋯S/S⋯H	16.6
H⋯N/N⋯H	4.3
H⋯O/O⋯H	3.2
O⋯C/C⋯O	0.7
O⋯S/S⋯O	0.6

**Table 3:** Experimental details

Crystal data	
Chemical formula	[Ni(NCS)_2_(C_15_H_22_N_2_O2)_2_]
*M*_r_	699.56
Crystal system, space group	Monoclinic, *P*2_1_/*c*
Temperature (K)	100
*a*, *b*, *c* (Å)	12.6406 (3), 10.1280 (3), 15.7458 (4)
β (°)	108.647 (3)
*V* (Å^3^)	1910.02 (9)
*Z*	2
Radiation type	Mo *K*α
μ (mm^−1^)	0.66
Crystal size (mm)	0.39 × 0.24 × 0.16

Data collection	
Diffractometer	Agilent SuperNova, Dual, Cu at zero, Atlas
Absorption correction	Multi-scan (*CrysAlis PRO*; Agilent, 2014 ▸)
*T*_min_, *T*_max_	0.772, 1.000
No. of measured, independent and observed [*I* > 2σ(*I*)] reflections	12439, 4575, 3961
*R*_int_	0.027
(sin θ/λ)_max_ (Å^−1^)	0.695

Refinement	
*R*[*F* ^2^ > 2σ(*F* ^2^)], *wR*(*F* ^2^), *S*	0.031, 0.073, 1.05
No. of reflections	4575
No. of parameters	210
H-atom treatment	H atoms treated by a mixture of independent and constrained refinement
Δρ_max_, Δρ_min_ (e Å^−3^)	0.33, −0.39

Computer programs: *CrysAlis PRO* (Agilent, 2014 ▸), *SHELXT* (Sheldrick, 2015*a* ▸), *SHELXL2018/1* (Sheldrick, 2015*b* ▸), *TITAN* (Hunter & Simpson, 1999 ▸), *Mercury* (Macrae *et al.*, 2020 ▸), *enCIFer* (Allen *et al.*, 2004 ▸), *PLATON* (Spek, 2020 ▸) and *publCIF* (Westrip 2010 ▸).

## supplementary crystallographic information

### Crystal data

**Table:**

[Ni(NCS)_2_(C_15_H_22_N_2_O2)_2_]	*F*(000) = 740
*M**_r_* = 699.56	*D*_x_ = 1.216 Mg m^−^^3^
Monoclinic, *P*2_1_/*c*	Mo *K*α radiation, λ = 0.71073 Å
*a* = 12.6406 (3) Å	Cell parameters from 6613 reflections
*b* = 10.1280 (3) Å	θ = 3.6–29.2°
*c* = 15.7458 (4) Å	µ = 0.66 mm^−^^1^
β = 108.647 (3)°	*T* = 100 K
*V* = 1910.02 (9) Å^3^	Rectangular block, blue
*Z* = 2	0.39 × 0.24 × 0.16 mm

### Data collection

**Table:**

Agilent SuperNova, Dual, Cu at zero, Atlas diffractometer	4575 independent reflections
Radiation source: Agilent SuperNova (Mo) X-ray Source	3961 reflections with *I* > 2σ(*I*)
Detector resolution: 5.1725 pixels mm^-1^	*R*_int_ = 0.027
ω scans	θ_max_ = 29.6°, θ_min_ = 3.2°
Absorption correction: multi-scan (CrysAlisPro; Agilent, 2014)	*h* = −17→17
*T*_min_ = 0.772, *T*_max_ = 1.000	*k* = −13→13
12439 measured reflections	*l* = −21→21

### Refinement

**Table:**

Refinement on *F*^2^	0 restraints
Least-squares matrix: full	Hydrogen site location: mixed
*R*[*F*^2^ > 2σ(*F*^2^)] = 0.031	H atoms treated by a mixture of independent and constrained refinement
*wR*(*F*^2^) = 0.073	*w* = 1/[σ^2^(*F*_o_^2^) + (0.0259*P*)^2^ + 0.7321*P*] where *P* = (*F*_o_^2^ + 2*F*_c_^2^)/3
*S* = 1.05	(Δ/σ)_max_ = 0.001
4575 reflections	Δρ_max_ = 0.33 e Å^−^^3^
210 parameters	Δρ_min_ = −0.38 e Å^−^^3^

### Special details

**Table:**

Geometry. All esds (except the esd in the dihedral angle between two l.s. planes) are estimated using the full covariance matrix. The cell esds are taken into account individually in the estimation of esds in distances, angles and torsion angles; correlations between esds in cell parameters are only used when they are defined by crystal symmetry. An approximate (isotropic) treatment of cell esds is used for estimating esds involving l.s. planes.
Refinement. One reflection with Fo >>> Fc was omitted from the final refinement cycles.

### Fractional atomic coordinates and isotropic or equivalent isotropic displacement parameters (Å^2^)

**Table:**

	*x*	*y*	*z*	*U*_iso_*/*U*_eq_	
Ni1	0.500000	0.000000	1.000000	0.01311 (7)	
O1	0.34390 (7)	0.04585 (10)	1.01346 (6)	0.0166 (2)	
C1	0.33197 (11)	−0.01165 (14)	1.07823 (9)	0.0159 (3)	
O2	0.25009 (8)	0.01215 (11)	1.11267 (7)	0.0210 (2)	
C2	0.16396 (11)	0.10284 (16)	1.06322 (10)	0.0208 (3)	
H2A	0.137728	0.078992	0.998872	0.025*	
H2B	0.193675	0.194018	1.069115	0.025*	
C3	0.06895 (11)	0.09413 (16)	1.10129 (10)	0.0207 (3)	
C4	−0.00906 (14)	0.19554 (19)	1.08242 (13)	0.0357 (4)	
H4	−0.000374	0.268997	1.047719	0.043*	
C5	−0.09990 (15)	0.1901 (2)	1.11410 (14)	0.0428 (5)	
H5	−0.153094	0.259680	1.100762	0.051*	
C6	−0.11294 (13)	0.0844 (2)	1.16464 (12)	0.0338 (4)	
H6	−0.174835	0.081002	1.186420	0.041*	
C7	−0.03610 (13)	−0.01634 (18)	1.18350 (11)	0.0277 (4)	
H7	−0.044911	−0.089213	1.218626	0.033*	
C8	0.05482 (12)	−0.01236 (16)	1.15143 (10)	0.0226 (3)	
H8	0.107027	−0.082965	1.164090	0.027*	
N2	0.39863 (10)	−0.10797 (13)	1.12383 (8)	0.0187 (3)	
H2N	0.3879 (13)	−0.1414 (17)	1.1681 (12)	0.022*	
N1	0.48528 (9)	−0.14754 (12)	1.09226 (8)	0.0156 (2)	
C9	0.52823 (11)	−0.26137 (15)	1.11779 (9)	0.0170 (3)	
C10	0.48835 (12)	−0.35599 (15)	1.17505 (10)	0.0194 (3)	
H10A	0.470958	−0.306629	1.223216	0.023*	
H10B	0.548117	−0.420395	1.203426	0.023*	
C11	0.38364 (14)	−0.42957 (18)	1.11758 (11)	0.0308 (4)	
H11A	0.321679	−0.365719	1.094963	0.037*	
H11B	0.398761	−0.469318	1.065186	0.037*	
C12	0.34816 (16)	−0.53771 (19)	1.17007 (13)	0.0375 (4)	
H12A	0.406779	−0.605036	1.188334	0.056*	
H12B	0.278687	−0.578254	1.132223	0.056*	
H12C	0.336205	−0.499402	1.223428	0.056*	
C13	0.62074 (12)	−0.30822 (16)	1.08489 (10)	0.0209 (3)	
H13A	0.618840	−0.257813	1.030547	0.025*	
H13B	0.608894	−0.402577	1.068126	0.025*	
C14	0.73513 (12)	−0.29144 (18)	1.15569 (12)	0.0290 (4)	
H14A	0.734359	−0.332963	1.212408	0.035*	
H14B	0.751004	−0.196209	1.167194	0.035*	
C15	0.82738 (14)	−0.3542 (2)	1.12562 (14)	0.0391 (5)	
H15A	0.814383	−0.449445	1.117866	0.059*	
H15B	0.899948	−0.338260	1.171121	0.059*	
H15C	0.826912	−0.314947	1.068597	0.059*	
N3	0.57102 (10)	0.12673 (13)	1.09988 (8)	0.0189 (3)	
C16	0.61615 (11)	0.21720 (15)	1.14054 (9)	0.0162 (3)	
S1	0.68148 (3)	0.34303 (4)	1.19984 (3)	0.02219 (10)	

### Atomic displacement parameters (Å^2^)

**Table:**

	*U*^11^	*U*^22^	*U*^33^	*U*^12^	*U*^13^	*U*^23^
Ni1	0.01222 (12)	0.01537 (14)	0.01307 (13)	−0.00022 (9)	0.00589 (10)	−0.00050 (10)
O1	0.0141 (4)	0.0200 (5)	0.0175 (5)	0.0017 (4)	0.0076 (4)	0.0030 (4)
C1	0.0132 (6)	0.0193 (8)	0.0164 (7)	−0.0004 (5)	0.0066 (5)	−0.0015 (6)
O2	0.0172 (5)	0.0278 (6)	0.0224 (5)	0.0092 (4)	0.0126 (4)	0.0091 (4)
C2	0.0168 (7)	0.0233 (8)	0.0227 (7)	0.0063 (6)	0.0069 (6)	0.0052 (6)
C3	0.0163 (7)	0.0258 (9)	0.0207 (7)	0.0031 (6)	0.0071 (6)	−0.0024 (6)
C4	0.0322 (9)	0.0337 (11)	0.0487 (11)	0.0130 (8)	0.0233 (8)	0.0116 (9)
C5	0.0314 (9)	0.0460 (12)	0.0597 (13)	0.0201 (9)	0.0269 (9)	0.0074 (10)
C6	0.0204 (8)	0.0495 (12)	0.0376 (9)	0.0033 (8)	0.0177 (7)	−0.0030 (9)
C7	0.0189 (7)	0.0404 (11)	0.0251 (8)	−0.0020 (7)	0.0087 (7)	0.0017 (7)
C8	0.0147 (7)	0.0306 (9)	0.0229 (8)	0.0023 (6)	0.0066 (6)	0.0004 (7)
N2	0.0181 (6)	0.0228 (7)	0.0201 (6)	0.0053 (5)	0.0128 (5)	0.0060 (5)
N1	0.0134 (5)	0.0196 (7)	0.0161 (6)	0.0022 (5)	0.0081 (5)	−0.0003 (5)
C9	0.0167 (6)	0.0184 (8)	0.0164 (6)	−0.0002 (6)	0.0060 (5)	−0.0006 (6)
C10	0.0228 (7)	0.0173 (8)	0.0209 (7)	0.0022 (6)	0.0109 (6)	0.0010 (6)
C11	0.0372 (9)	0.0278 (10)	0.0280 (8)	−0.0102 (7)	0.0110 (7)	−0.0019 (7)
C12	0.0428 (10)	0.0303 (10)	0.0407 (10)	−0.0142 (8)	0.0154 (9)	−0.0007 (8)
C13	0.0241 (7)	0.0185 (8)	0.0244 (7)	0.0047 (6)	0.0139 (6)	0.0030 (6)
C14	0.0216 (7)	0.0288 (10)	0.0385 (9)	0.0033 (7)	0.0124 (7)	0.0056 (8)
C15	0.0280 (8)	0.0395 (11)	0.0571 (12)	0.0144 (8)	0.0237 (9)	0.0214 (9)
N3	0.0196 (6)	0.0213 (7)	0.0167 (6)	−0.0013 (5)	0.0072 (5)	−0.0016 (5)
C16	0.0157 (6)	0.0195 (8)	0.0162 (6)	0.0031 (6)	0.0090 (5)	0.0028 (6)
S1	0.02393 (19)	0.0196 (2)	0.0279 (2)	−0.00579 (15)	0.01512 (16)	−0.00852 (16)

### Geometric parameters (Å, º)

**Table:**

Ni1—N3	2.0059 (13)	N2—H2N	0.824 (17)
Ni1—N3^i^	2.0059 (12)	N1—C9	1.2829 (19)
Ni1—O1^i^	2.1028 (9)	C9—C13	1.4994 (18)
Ni1—O1	2.1028 (9)	C9—C10	1.5086 (19)
Ni1—N1^i^	2.1332 (12)	C10—C11	1.536 (2)
Ni1—N1	2.1332 (12)	C10—H10A	0.9900
O1—C1	1.2249 (17)	C10—H10B	0.9900
C1—O2	1.3350 (15)	C11—C12	1.523 (2)
C1—N2	1.3380 (19)	C11—H11A	0.9900
O2—C2	1.4467 (17)	C11—H11B	0.9900
C2—C3	1.5066 (18)	C12—H12A	0.9800
C2—H2A	0.9900	C12—H12B	0.9800
C2—H2B	0.9900	C12—H12C	0.9800
C3—C8	1.381 (2)	C13—C14	1.527 (2)
C3—C4	1.389 (2)	C13—H13A	0.9900
C4—C5	1.392 (2)	C13—H13B	0.9900
C4—H4	0.9500	C14—C15	1.530 (2)
C5—C6	1.375 (3)	C14—H14A	0.9900
C5—H5	0.9500	C14—H14B	0.9900
C6—C7	1.374 (2)	C15—H15A	0.9800
C6—H6	0.9500	C15—H15B	0.9800
C7—C8	1.396 (2)	C15—H15C	0.9800
C7—H7	0.9500	N3—C16	1.1582 (19)
C8—H8	0.9500	C16—S1	1.6386 (16)
N2—N1	1.3985 (15)		
			
N3—Ni1—N3^i^	180.00 (7)	N1—N2—H2N	122.8 (12)
N3—Ni1—O1^i^	91.21 (4)	C9—N1—N2	116.57 (11)
N3^i^—Ni1—O1^i^	88.79 (4)	C9—N1—Ni1	136.26 (9)
N3—Ni1—O1	88.79 (4)	N2—N1—Ni1	106.86 (8)
N3^i^—Ni1—O1	91.21 (4)	N1—C9—C13	118.34 (12)
O1^i^—Ni1—O1	180.0	N1—C9—C10	124.70 (12)
N3—Ni1—N1^i^	88.34 (5)	C13—C9—C10	116.88 (13)
N3^i^—Ni1—N1^i^	91.66 (5)	C9—C10—C11	110.23 (12)
O1^i^—Ni1—N1^i^	78.29 (4)	C9—C10—H10A	109.6
O1—Ni1—N1^i^	101.71 (4)	C11—C10—H10A	109.6
N3—Ni1—N1	91.66 (5)	C9—C10—H10B	109.6
N3^i^—Ni1—N1	88.34 (5)	C11—C10—H10B	109.6
O1^i^—Ni1—N1	101.71 (4)	H10A—C10—H10B	108.1
O1—Ni1—N1	78.29 (4)	C12—C11—C10	112.14 (14)
N1^i^—Ni1—N1	180.0	C12—C11—H11A	109.2
C1—O1—Ni1	110.38 (9)	C10—C11—H11A	109.2
O1—C1—O2	124.76 (13)	C12—C11—H11B	109.2
O1—C1—N2	124.70 (12)	C10—C11—H11B	109.2
O2—C1—N2	110.53 (12)	H11A—C11—H11B	107.9
C1—O2—C2	116.34 (11)	C11—C12—H12A	109.5
O2—C2—C3	107.88 (12)	C11—C12—H12B	109.5
O2—C2—H2A	110.1	H12A—C12—H12B	109.5
C3—C2—H2A	110.1	C11—C12—H12C	109.5
O2—C2—H2B	110.1	H12A—C12—H12C	109.5
C3—C2—H2B	110.1	H12B—C12—H12C	109.5
H2A—C2—H2B	108.4	C9—C13—C14	111.94 (12)
C8—C3—C4	119.14 (13)	C9—C13—H13A	109.2
C8—C3—C2	122.57 (13)	C14—C13—H13A	109.2
C4—C3—C2	118.26 (14)	C9—C13—H13B	109.2
C3—C4—C5	120.33 (17)	C14—C13—H13B	109.2
C3—C4—H4	119.8	H13A—C13—H13B	107.9
C5—C4—H4	119.8	C13—C14—C15	111.43 (15)
C6—C5—C4	120.24 (16)	C13—C14—H14A	109.3
C6—C5—H5	119.9	C15—C14—H14A	109.3
C4—C5—H5	119.9	C13—C14—H14B	109.3
C7—C6—C5	119.73 (14)	C15—C14—H14B	109.3
C7—C6—H6	120.1	H14A—C14—H14B	108.0
C5—C6—H6	120.1	C14—C15—H15A	109.5
C6—C7—C8	120.48 (16)	C14—C15—H15B	109.5
C6—C7—H7	119.8	H15A—C15—H15B	109.5
C8—C7—H7	119.8	C14—C15—H15C	109.5
C3—C8—C7	120.07 (15)	H15A—C15—H15C	109.5
C3—C8—H8	120.0	H15B—C15—H15C	109.5
C7—C8—H8	120.0	C16—N3—Ni1	163.23 (11)
C1—N2—N1	116.66 (11)	N3—C16—S1	178.75 (14)
C1—N2—H2N	120.5 (12)		
			
Ni1—O1—C1—O2	169.27 (11)	O1—C1—N2—N1	−2.4 (2)
Ni1—O1—C1—N2	−11.39 (18)	O2—C1—N2—N1	177.07 (12)
O1—C1—O2—C2	6.7 (2)	C1—N2—N1—C9	−160.49 (13)
N2—C1—O2—C2	−172.72 (12)	C1—N2—N1—Ni1	14.19 (15)
C1—O2—C2—C3	168.23 (12)	N2—N1—C9—C13	179.49 (12)
O2—C2—C3—C8	−19.5 (2)	Ni1—N1—C9—C13	6.9 (2)
O2—C2—C3—C4	162.63 (15)	N2—N1—C9—C10	3.0 (2)
C8—C3—C4—C5	0.4 (3)	Ni1—N1—C9—C10	−169.67 (10)
C2—C3—C4—C5	178.27 (17)	N1—C9—C10—C11	79.24 (18)
C3—C4—C5—C6	0.2 (3)	C13—C9—C10—C11	−97.34 (15)
C4—C5—C6—C7	−0.3 (3)	C9—C10—C11—C12	173.28 (14)
C5—C6—C7—C8	−0.3 (3)	N1—C9—C13—C14	101.19 (16)
C4—C3—C8—C7	−0.9 (2)	C10—C9—C13—C14	−82.00 (17)
C2—C3—C8—C7	−178.70 (15)	C9—C13—C14—C15	173.33 (13)
C6—C7—C8—C3	0.8 (3)		

Symmetry code: (i) −*x*+1, −*y*, −*z*+2.

### Hydrogen-bond geometry (Å, º)

Cg is the centroid of the C3–C8 phenyl ring.

**Table:**

*D*—H···*A*	*D*—H	H···*A*	*D*···*A*	*D*—H···*A*
N2—H2*N*···S1^ii^	0.824 (17)	2.507 (17)	3.2830 (12)	157.3 (16)
C8—H8···S1^ii^	0.95	2.94	3.7080 (16)	139
C10—H10*A*···S1^ii^	0.99	3.00	3.9059 (14)	154
C10—H10*B*···S1^iii^	0.99	2.94	3.8464 (15)	153
C13—H13*A*···O1^i^	0.99	2.35	3.1783 (18)	141
C2—H2*A*···*Cg*3^iv^	0.99	2.72	3.6041 (17)	149

Symmetry codes: (i) −*x*+1, −*y*, −*z*+2; (ii) −*x*+1, *y*−1/2, −*z*+5/2; (iii) *x*, *y*−1, *z*; (iv) −*x*, −*y*, −*z*+2.
